# Supplementary material for: Evaluation of masticatory muscles in temporomandibular joint disorder patients using quantitative MRI fat fraction analysis—Could it be a biomarker?
Source: PLoS One. 2024 Jan 19;19(1):e0296769. doi: 10.1371/journal.pone.0296769 (PMC10798479; doi:10.1371/journal.pone.0296769)
Supplement: Attachment — Submitted filename: Response to Reviewers.docx [file pone.0296769.s002.docx]

Reviewers' comments:

**Journal Requirements:**

Please review your reference list to ensure that it is complete and correct. If you have cited papers that have been retracted, please include the rationale for doing so in the manuscript text, or remove these references and replace them with relevant current references. Any changes to the reference list should be mentioned in the rebuttal letter that accompanies your revised manuscript. If you need to cite a retracted article, indicate the article’s retracted status in the References list and also include a citation and full reference for the retraction notice.

**Reply 1:** We reviewed the reference list to ensure that it is complete and correct. We did not cite the retracted paper and modified it to fit the form.

**Changes in the text:** We highlighted the changed parts.

6. Schiffman E, Ohrbach R, Truelove E, Look J, Anderson G, Goulet JP, et al. Diagnostic Criteria for Temporomandibular Disorders (DC/TMD) for Clinical and Research Applications: recommendations of the International RDC/TMD Consortium Network* and Orofacial Pain Special Interest Group†. J Oral Facial Pain Headache. 2014;28(1):6-27.

**Reviewer 1:**

**Comment 1:** Thank you for your reply. For the most part, the authors responded correctly to my comments.

Except for one point. The authors did not add the effect size to the results. The effect size is not the same as the effect size given to the sample size calculation. Please provide the effect size for each outcome. doi: 10.4300/JGME-D-12-00156.1

**Reply 1:** We added the effect size to the results based on your advice. (See page 8, lines 138, 144 and 147-149).

**Changes in the text:** We highlighted the changed parts.

**Table 3. Fat fraction (%) of the normal group (n = 19)**

|  | **Right side** | **Left side** | ***p*-value** | ***Effect size***  ***(Cohen’s d)*** |
| --- | --- | --- | --- | --- |
| **Masseter muscle** | 2.95 ± 0.96 | 2.92 ± 1.12 | 0.857 | -0.042 |
| **Medial pterygoid muscle** | 2.04 ± 1.18 | 1.83 ± 0.68 | 0.365 | -0.213 |
| **Lateral pterygoid muscle** | 2.88 ± 1.13 | 2.92 ± 1.24 | 0.844 | 0.046 |

**Table 4. Fat fraction (%) of the TMD group (n = 46)**

|  | **Normal side** | **Disc displacement side** | ***p*-value** | ***Effect size***  ***(Cohen’s d)*** |
| --- | --- | --- | --- | --- |
| **Masseter muscle** | 3.86 ± 1.96 | 3.68 ± 1.65 | 0.303 | -0.154 |
| **Medial pterygoid muscle** | 2.74 ± 1.22 | 2.48 ± 1.15 | 0.089 | -0.256 |
| **Lateral pterygoid muscle** | 2.60 ± 1.23 | 2.77 ± 1.41 | 0.323 | 0.147 |

“*Within the TMD subgroups, only the masseter muscle in the subgroup without clenching had a statistically significantly lower FF value on the disc displacement side (3.94 ± 1.61) than on the normal side (4.52 ± 2.24) (p < 0.05, Cohen’s d = -0.502) (Fig. 2, Fig. 3).”*
